# Role of human adipose-derived stem cells (hADSC) on TGF-β1, type I collagen, and fibrosis degree in bladder obstruction model of Wistar rats

**DOI:** 10.1186/s12894-022-01019-2

**Published:** 2022-04-24

**Authors:** Safendra Siregar, Mochamad Sri Herlambang, Muhammad Reza, Akhmad Mustafa, Dicky Stefanus

**Affiliations:** Department of Urology, Faculty of Medicine Universitas Padjadjaran, Hasan Sadikin General Hospital Bandung, Jln. Pasteur No. 38, Bandung, Indonesia

**Keywords:** Adipose-derived stem cells, TGF β1, Collagen type 1, Degree of fibrosis

## Abstract

**Introduction:**

Bladder outlet obstruction (BOO) was caused by a series of histological and biochemical changes in the bladder wall, through the inflammation process in the bladder wall, hypertrophy and fibrosis. ADSC has an important role in bladder regeneration.

**Methods and materials:**

This study was an experimental randomized study using male Wistar rats which were monitored at 2 and 4 weeks to determine the effect of ADSC therapy on TGF-β1 type I collagen, and degree of fibrosis.

**Result:**

Rats were divided into 5 groups. In the week 2 BOO group, 1 sample included in the category of moderate fibrosis, 1 sample that was given ADSC with mild fibrosis category, 3 samples included in severe fibrosis category, 3 samples that were given ADSC included in the category of moderate fibrosis. The concentration of TGF-β1 in the hADSC therapy group was significantly lower than the control group at the 2nd and 4th week of monitoring (p2 = 0.048, p4 = 0.048), and also with more type I collagen on 2nd and the 4th week (p2 = 0.048, p4 = 0.048).

**Conclusion:**

ADSC therapy can reduce the concentration of TGF-β1, type I collagen, and degree of fibrosis in the male Wistar BOO model.

## Introduction

Bladder outlet obstruction (BOO) is one of the most common problem in urology. BOO most commonly caused by benign prostatic obstruction (BPO) in adult, while in children BOO is mostly due to a congenital anomaly such as Posterior Urethral Valve (PUV) [1, 2]. Chronically obstructed bladder underwent series of histological and biochemical changes in the bladder wall, through the inflammation process in the bladder wall, leading to hypertrophy and fibrosis at the decompensation stage [1–3].

BOO may lead to functional impairment of bladder due to fibrosis that preceded by inflammation. The fibrosis process is characterised by a disarrangement in the organisation of extracellular matrix (ECM) due to increased synthesis of type I collagen, increased fibroblasts and gross architectural change of the bladder. This changes causes smooth muscle dysfunction and decreased bladder compliance [4]. Metabolic syndrome is also known for the cause of chronic prostatic inflammation through the deposition of elastin and collagen which lead to periurethral fibrosis and bladder outlet obstruction. (sumber baru) The renin-angiotensin system (RAS) and TGF-β are known to be mediators of tissue fibrosis in a number of organ systems, however, their function in bladder fibrosis are poorly understood [5].

Many studies had been conducted to overcome the fibrosis of the bladder, with the aim to ameliorate bladder dysfunction such as antifibrotic agents pirfenidone, N-acetylcysteine and relaxin-2 [6]. Although many therapeutic options for bladder dysfunction have previously been developed, improvement in symptoms of urinary dysfunction has not been fully achieved as expected. In recent years, many studies have shown the potential anti-fibrotic effect of adipose tissue derived stem cells (ADSC) in many animal models. ADSC are type of mesenchymal stem cells, that exhibit tissue plasticity and potency to produce various secretome that promote healing in vivo. Currently, ADSC have been used as a therapy for injured organ regeneration in animal models and in a few clinical trials [7–9]. ADSC also proved to ameliorate collagen deposition in various organs [10]. This study aims to evaluate the effect of human ADSC administration on fibrosis process inhibition in bladder outlet obstruction model of Wistar rats through tissue TGF-β_1_, type I collagen, and fibrosis degree.

## Material and methods

### Human ADSCs isolation and characterization

Human adipose tissue was collected from the fat of an adult male or female who had undergone prior surgery and had previously signed an informed permission form allowing his or her fat to be donated for research. Cell characterisation and transplantation were performed on this one-of-a-kind batch of cells isolated from a single fat donor. To promote better expansion, cells were collected and seeded till passage 3. Adult stem cells and embryonic stem cells are two types of stem cells. CD44 (+), CD45 (−), CD 73 (+), CD90 (+), and CD 105 (+) were used to describe isolated cells using a flow cytometry test. A total of 22.5 × 106 cells were collected, which were subsequently used in MEM in 1 cc syringes containing 1.0 × 106 cells. The protocol was authorized by the Universitas Airlangga Surabaya's Research and Ethical Committee, which also approved the fat collection method. We employed ADSC from adult human adipose tissue in this investigation for a number of reasons; (1) Sufficient source of cells from liposuction aspirate (2) Because we plan to employ human ADSC in human patients in the future, we first used human ADSC in rats, because human ADSC do not express MHC class-II and hence do not cause a xenogenic immune response. All tissue harvesting, cell isolation, culture, and characterization were completed at Dr. Soetomo General Hospital Surabaya's Tissue and Cell Bank with sample number 33.E/MSC/Penelitian/AdiposeSwt 01/090519 and certified acceptable for application.

### Partial bladder obstruction model sample

T A partial bladder obstruction model was created using a retropubic technique. The bladder was revealed after a 2-cm lower midline vertical incision. After that, the prostate was lifted to reach the bladder neck area. The seminal vesicle and ureters were not compromised since the prostate urethra was isolated. On the prostatic urethral surface, a sterile metal bar with a 0.91-mm diameter was put, and a 3–0 polypropylene suture was used to connect the prostatic urethra and the bar together. After the suture was secured, the bar was removed, partially obstructing the prostatic urethra. After surgery, the skin was closed with 4–0 nylon sutures, the incision was cleansed with povidone iodine, and the rat was placed in a cage under a heating lamp [11].

**Fig. 1:**
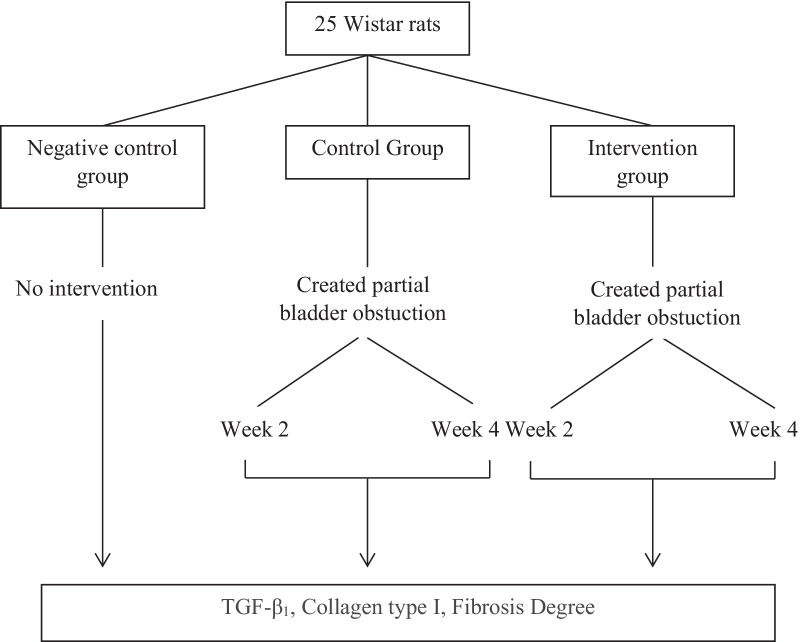
Research flow

Twenty-five Wistar rats were included (Fig. 1). All samples were separated into five groups, with two groups consisting of five male Wistar rats with partial bladder blockage models who were not given hADSC therapy and were injected intravenously with 1 cc of hADSC solvent immediately after surgery. After 2 and 4 weeks of observation, these rats were killed (group I and II, control group). Five rats with a partial bladder blockage model were administered intravenous 1.0 × 106 cells hADSC injections immediately after model construction and were terminated after 2 and 4 weeks of observation (group III and IV, treatment group). The last group consists of five rats who have not received any treatment or have not been exposed to any model (group V, negative control group). Each rat was slaughtered and then had a cystectomy for TGF-1 and collagen type I. The tissue was subsequently treated from various places in bladder and yielding 1 g of tissue extract from for measuring TGF-1 and collagen type I. Type 1 collagen in the tissue would stick to the plate which had been coated with type 1 collagen antibody. Prior to processing, the tissue was rinsed using Phosphate Buffer Saline (PBS) solution (pH 7.4) to remove excess blood and tissue. The tissue was then smoothed and homogenized in PBS solution (pH 7.4) with a glass homogenizer on ice. Tissue could be thawed at 2–8 °C or frozen at − 20 °C if it was to be stored beforehand. Prior to the procedure, the sample tissue was allowed to reach room temperature and then centrifuged at 2000–3000 RPM for 20 min. ELISA (enzyme-linked immunosorbent assay) measurements were performed to evaluate the TGF-1 and collagen type 1. (FineTest^®^ Rat Col1 ELISA kit range 0.156-10 ng/ml and FineTest^®^ TGF-1 ELISA kit range 31.25–2000 pg/ml).

The tissue samples were fixed in a 10% formaldehyde solution. The sample is then processed and made into paraffin blocks. Paraffin-embedded tissue was sliced and placed on an object-glass, followed by deparaffinization and rehydration. Hydrogen peroxide was administered for 10 min to eliminate endogenous peroxidase activity. The sample was then rinsed using PBS-Triton X 100 (Tx). Hematoxylin–eosin (HE) staining was performed to assess structural changes in the bladder tissue. To determine smooth muscle and collagen, cross-sections were stained with Masson's trichrome (MT) using the MT staining kit. Smooth muscle areas (red stains) and collagen (blue-green stains) were evaluated in 200× magnification images of tissue using a microscope. The degree of fibrosis in mice was classified as no fibrosis (not found/0 fibroblasts), mild fibrosis (25 fibroblasts), moderate fibrosis (25–50 fibroblasts), and severe fibrosis (> 50 fibroblasts) based on inflammation and the degree of fibrosis. If the p value is less than 0.05, the difference is considered significant [12].

All of the animal models in this investigation were kept in solid plastic cages, with five rats per cage. The rats were given free access to conventional rodent diet and water. This work was carried out in the Biochemical and Bioscience Laboratory at Brawijaya University Malang and was approved by the ethical committee. According to William Russel and Rex Burch, all study objects were treated in accordance with animal welfare guidelines. Animal studies are reported in compliance with the ARRIVE guidelines. All experimental protocols were approved by Padjadjaran University. For statistical analysis, values are expressed as mean ± SD (ng/ml). Groups were compared with independent-sample T test. Values of *p* < 0.05 were considered statistically significant. SPSS statistics were used for the statistical analysis.

## Results

**Fig. 2:**
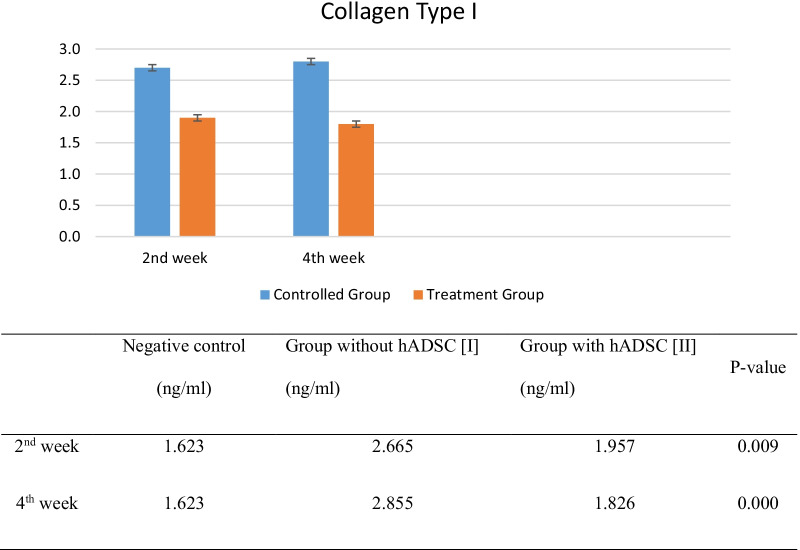
Effects of ADSC to collagen type I

Twenty six Wistar rats were divided into 5 groups. They were checked for collagen type 1 and TGF-β_1_. Result of collagen type I examination using ELISA method showed that the collagen type I concentration was higher in group I than group II in 2nd week and 4th week of observation. From statistical analysis using independent sample t-test, there were significant differences in collagen type I concentration between group I and group II, in 2nd week of observation (*p* = 0.009) and 4th week of observation (*p* = 0.000) (Fig. 2).

**Fig. 3:**
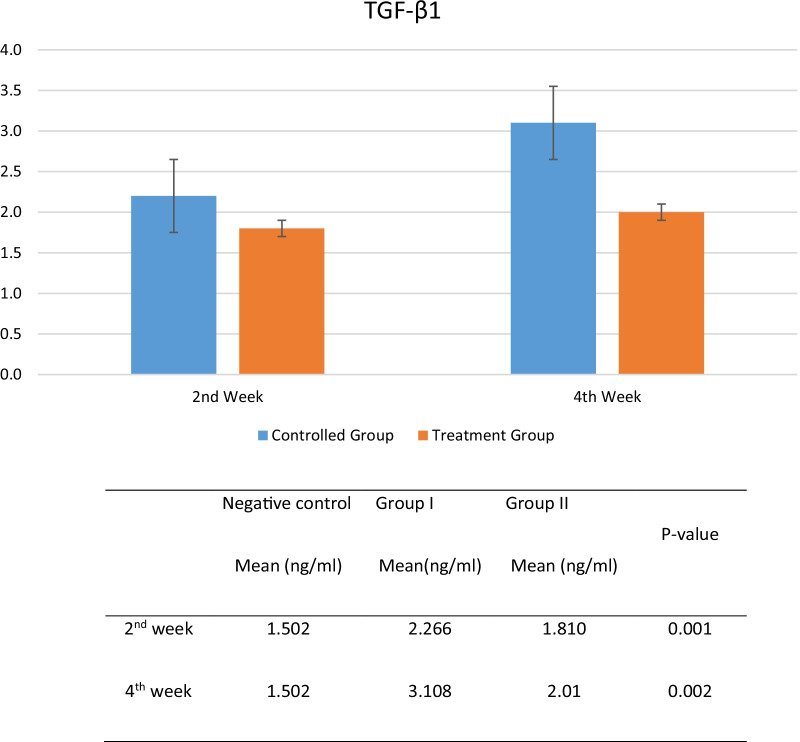
Effects of ADSC to TGF-β1

Result of TGF-β_1_ examination using ELISA method showed that there was a concentration difference between group I (control) and group II (hADSC treatment). TGF-β_1_ concentration was higher in control group, compared to treatment group in 2nd and 4th week of observation. Statistical analysis using independent sample T-test comparing group I and group II at the time of observation while group III will be excluded from comparison and only act as tissue concentration baseline. From statistical analysis, there were significant differences in TGF-β_1_ concentration between group I and group II, in 2nd week of observation (*p* = 0.001) and 4th week of observation (*p* = 0.002). Even though there was also a tendency in TGF-β_1_ concentration increase in group II, but the progression was higher in group I (Fig. 3).

Based on Table 1, 1 sample in the fibrosis group of pBOO week 2 was categorized as mild fibrosis, 1 sample in the fibrosis group of pBOO week 2 with ADSCs was categorized as mild fibrosis. 3 samples in the fibrosis group of pBOO week 2 were categorized as severe, and 3 samples in the fibrosis group of pBOO week 2 with ADSCs were categorized as moderate.

**Table 1:** Fibrosis analysis on the pBOO week 2 and fibrosis on the pBOO week 4 with ADSCs

Fibrosis degree	Week 2 without hADSC	Week 2 with hADSC	Week 4 without hADSC	Week 4 with hADSC	*p* value
Mild	0	1	0	2	0.046
Moderate	1	3	2	2	
Severe	3	0	2	0	
Total	4	4	4	4	

**Fig. 4:**
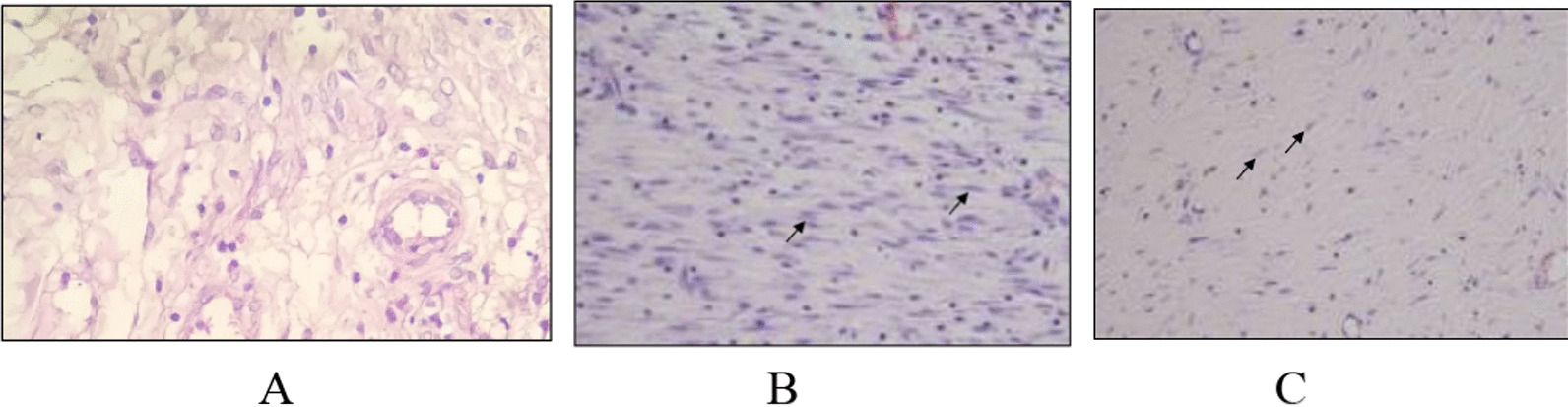
**A** Bladder fibrosis of the control group. **B** Fibrosis in the pBOO without hADSC. **C** Fibrosis in the pBOO with hADSC

Two samples in the fibrosis group of pBOO week 4 were categorized as moderate fibrosis, 2 samples in the fibrosis group with pBOO week 4 with ADSCs were categorized as mild fibrosis. 2 samples in the fibrosis group of pBOO week 4 were categorized as severe fibrosis, and 2 samples in the fibrosis group of pBOO week 4 with ADSCs were categorized as moderate fibrosis. To determine the effect of ADSCs on fibrosis pBOO, a chi-square analysis was carried out with a statistical result *p* value of 0.046 (Fig. 4).

## Discussion

BOO causes a series of histological and biochemical changes in the bladder wall, through the initial process of inflammation in the bladder wall, tissue hypoxia, hypertrophy, tissue compensation and eventual fibrosis at the decompensation stage [6, 13]. Inflammation process in bladder causes histopathological changes which are characterized by an increase in the organization of extracellular matrix (ECM), one of which is collagen type I produced by fibroblast. The production of collagen type I is mediated by increase of TGF-β1 that upregulated due to inflammation [9–11, 14]. MSC successfully prevented the growth of fibrotic tissue in experimental or preclinical studies [15]. MSC transplantation was also able to inhibit the formation of fibrotic tissue and to restore the functions of previously disturbed organs, such as the heart, liver, and lungs.

This study used Wistar rats model of partial bladder obstruction. Fibrosis cause decrease of TGF-β1 in partial obstruction. We observe TGF-β1 and type 1 collagen that decrease due to fibrosis. Our study showed decrease in TGF-β1 and type 1 collagen in population treated with ADSC compared with sham population. Stem cell injection has been showed for reducing fibrosis in other organs, such as liver, vascular, and kidney [9, 14, 16]. Based on this research, we can conclude that ADSC can be used to provide the same benefits.

ADSC can be used with various mechanism. Through homing process mechanism, stem cells had an ability to actively navigate in circulation through endothelium of different organ. This mechanism is mediated by specific receptor or ligand expression that facilitate movement, adhesion, and infiltration of stem cell into injured organ, similar to how leukocyte migrate into inflamed tissue. TGF-β1 activation showed strong chemotaxis to monocyte, neutrophil, fibroblast, and mesenchyme. These cells can produce and secrete more TGF-β1 that react to fiber cell and induce collagen type I and type III, fibronectin, elastin protein, intergin, proteoglycan, and other extracellular matrix component synthesis [15]. Study by Zhang et al. showed that increase TGF-β1 receptor on membrane cell can increase ADSC sensitivity to TGF-β1 and faster ADSC differentiation [16].

Study conducted by Zhang et al. showed that in BOO models, bladder dysfunction treated with ADSCs and MPCs showed high levels of both smooth muscle actin (SMA) and smoothelin expression. Smooth muscle regeneration was mainly achieved by paracine factors or by direct differentiation into new smooth muscle tissue by injecting SCs. The interaction of cell surface receptors, ligands, and short-range-acting molecules between differentiated and undifferentiated cells significantly contributes to stem cell differentiation [17].

Wang et al. found that administration of MSC in mice resulted in inhibition of collagen buildup, which could *improve* bladder function. Specifically for the use of ADSC, the same results were found, namely in the form of expression of cells associated with significant proliferation in healing smooth muscles. Wang et al. found that not only morphological regeneration can occur more quickly, but also bladder function is improved in rats given by ADSC compared to control [18]. This study found that by administering ADSC, the number of fibroblasts could decrease. The impact of bladder damage that causes urinary disorders occurs due to changes in the composition and function of the bladder. Physiological differentiation can reduce the amount of connective tissue and increase smooth muscle tissue. The same study also found increased urinary function after ADSC administration which was higher than the control group. Decreased ratio of connective tissue and smooth muscle is one possibility that ADSC can be used in BOO cases to reduce the degree of fibrosis thus reducing the bladder outlet obstruction.

This study found a reduction of fibroblast amount with ADSCs administration (measured by histopathological studies). A significant reduction was observed when comparing the healthy control group with pBOO group with the same duration. Bladder injury, which caused urinary problems, occurred due to bladder compositional and functional changes. Tremp et al. found that mice with bladder obstruction had a thinner bladder wall (434.7 ± 39.0 μm compared to 815.8 ± 177 μm, *p* = 0.0067) compared to the control group [19]. A higher ratio of connective tissue and smooth muscle was also found with the obstruction compared to the normal control group (0.6 ± 0.05 vs. 0.47 ± 0.12). ADSCs administration in the intervention group caused a reduction of connective tissue and smooth muscle ratio compared to the group with bladder obstruction (0.36 ± 0.12 vs. 0.6 ± 0.05). This reduction of connective tissue was one of the signs of fibrosis reduction in cases with bladder obstruction. The available literature regarding the impact of ADSCs administration on fibrosis is still limited. However, the studies found that ADSC stem cells administration could induce cell differentiation in pBOO, particularly in fibrotic tissues. Physiological differentiation could reduce the amount of connective tissue and increase smooth muscle tissues. The same study also found an increased bladder function after higher ADSCs administration compared to the control group. The reduced ratio of connective tissue and smooth muscle (near the normal limits) is a possibility that ADSCs could be utilized in pBOO cases to reduce the degree of fibrosis.

The strength of this study was that it could be used as a reference for further studies to determine the effect of ADSCs in reducing bladder fibrosis levels due to chronic partial obstruction. ADSCs could be the new potential therapy in the future, as we know many stem cell-based therapy will be furtherly improved with the development of medical technology. Our hope for further study using ADSCs is the use of ADSCs intravesical injection in patients with severe BOO to prevent the development of the fibrotic process.

The limitations of this study included the limited sample size and time and the usage of human mesenchymal stem cells. The process of fibrosis may take longer than 4 weeks and our study limitation is we cannot evaluate the fibrotic process longer than 4 weeks. Further research study to evaluate the fibrotic process longer than 4 weeks may be needed. Evaluation of fibrosis using urodynamic studies can be an additional evaluation in the further study for multivariate analysis.

## Conclusion

Administration of ADSC therapy can reduce TGF-β1, collagen type I, and fibrosis degree in BOO models of male Wistar rats.

## Data Availability

The data described in this article can be freely and openly accessed at: https://drive.google.com/drive/folders/16xPPIuMocGJoXzdC1GAwRCvyhGEjodoz?usp=sharing.
